# Biodiversity Research in Central America: A Regional Comparison in Scientific Production Using Bibliometrics and Democracy Indicators

**DOI:** 10.3389/frma.2022.898818

**Published:** 2022-07-14

**Authors:** Jonathan A. Morales-Marroquín, Regina Solis Miranda, José Baldin Pinheiro, Maria Imaculada Zucchi

**Affiliations:** ^1^Genetics and Molecular Biology Department, Biology Institute, University of Campinas (UNICAMP), Campinas, Brazil; ^2^School of Languages and Cultures, University of Sheffield, Sheffield, United Kingdom; ^3^Department of Genetics, “Luiz de Queiroz” College of Agriculture, University of São Paulo, Piracicaba, Brazil; ^4^Unidade Regional de Pesquisa e Desenvolvimento (APTA), Secretaria de Agricultura, São Paulo, Brazil

**Keywords:** science mapping, biological diversity, species records, political corruption, Latin America, GBIF, climate change, state capture

## Abstract

Central America science production on biodiversity topics is important in planning future adaptive and conservation policies in a climate-related risk region that is considered a biodiversity hotspot but has the lowest Human Development Index of Latin America. Science production on biodiversity is related to geo-referenced species occurrence records, but the accessibility depends on political frameworks and science funding. This paper aims at foregrounding how the democratic shifts throughout the years have had an impact on science production on biodiversity research, and species records. For this exploration we developed a novel systematic scientometric analysis of science production on biodiversity topics, we used Bio-Dem (open-source software of biodiversity records and socio-political variables) and briefly analyzed the history—from 1980 to 2020—of Guatemala, El Salvador, Honduras, Nicaragua, Costa Rica, and Panama. With a data set of 16,304 documents, our analysis shows the significant discrepancies between the low science production of Central American Northern countries (Guatemala, El Salvador, Honduras, and Nicaragua), the prolific production from the Southern (Costa Rica and Panama), and how this relates to democratic stability. Scientific production tends to be more abundant when democratic conditions are guaranteed. The state capture phenomenon and colonial-rooted interactions worldwide have an effect on the conditions under which science is being produced in Central America. Democracy, science production, funding, and conservation are core elements that go hand in hand, and that need to be nourished in a region that struggles with the protection of life and extractive activities in a climate change scenario.

## Introduction

The Central American region has nuances in the development of its young democracies. Historical events such as colonial invasions, civil wars and democratic transitions have shaped the current state of governance, public policies, land use, natural and economical resource administration. On a global scale, Guatemala, El Salvador, Honduras, Nicaragua, and Panama ranked in the lowest positions of the Human Development Index for Latin America, on the contrary to Costa Rica which ranked amongst the top five (United Nations Development Programme, 2020). The investment in biodiversity research, embedded in academic and political efforts, also expresses these democratic and development frames (Barlow et al., 2018; Legagneux et al., 2018; Zizka et al., 2021). Science in Central America has had to navigate these contexts to generate funding opportunities to provide more data for public policy decision-making processes.

Central America harbors 5–12% of the planet's biodiversity, it is known as a hyperdiversity hotspot in the Neotropical region. This area is considered, for instance, one of the top five most diverse regions for vascular plants. It is a key area for understanding ecological, evolutionary and human demographic processes linked to the Tropical Rainforest species, because it connects North and South America (Barthlott et al., 2007; Kohlman et al., 2010; Meyer, N. F. et al., 2015; Eiserhardt et al., 2017; Barlow et al., 2018; Cano et al., 2022). The dynamic landscape of Central America had a direct impact on the diversification and colonization of biota in the isthmus, thus creating new niches. Despite the region's potential for investigation, little attention has been given in comparison to other Neotropical regions. Maybe, we asked ourselves, this lack of research has a reason involving democratic instability.

Tracking species records and science production on biodiversity topics is a way to estimate how much a country invests in understanding its natural resources. Central American countries have the lowest science funding of all Latin America, fostering a brain drain phenomenon (Bonilla, 2018; Bonilla and Serafim, 2021). The knowledge gaps compromise our ability to describe existing biodiversity and make accurate predictions that could support decisions in regards to climate change scenarios. This is observed in the great difference between the countries that have state scientific support, which are also the ones presenting better democratic indicators, versus those who don't and the number of species records they present (Zizka et al., 2021).

Species occurrence data is not updated in certain regions and habitats near conflict areas. Biodiversity inventories are more complete and comprehensive near locations where access, infrastructure and security is granted (Hortal et al., 2015; Meyer, C. et al., 2015; Daru et al., 2018). There are also historical patterns and colonization processes that modified the inventorying of biodiversity and its research, where endangered species and conservation projects could be affected (Eichhorn et al., 2020; Rydén et al., 2020). The geographic distribution of species and their link to human activities is essential in understanding commodity production, furthermore having a direct association with agriculture, health and social dynamics (Clement et al., 2004). Considering that Central American core commodities are tied with agrobusiness, biodiversity research is key in the construction of guidelines for regional development.

This study explores three elements of the Central American democracies—freedom of expression, political corruption, and polyarchy—in light of weak democratic transitions which led to a state capture phenomenon. Hellman and Kaufmann (2001) define state capture as “the efforts of firms to shape the laws, policies, and regulations of the state to their advantage by providing illicit private gains to public officials.” This phenomenon, also referred to as democracy privatization, names the influence that individuals, organizations or companies have upon the institutions and state policies in order to push for their own interests and against the population's wellbeing. The capture operates through mechanisms such as fiscal evasion, bribes to push for tailored laws, social leaders' criminalization, lobbying, financing political campaigns, revolving doors, investment in media, judicial capture, and violation of social, cultural and environmental rights. Therefore, state capture as a form of systemic corruption weakens democracy and erodes the possibility of research development via solid academic public institutions.

Biased data in species records or science production, or the lack of it, could limit the use of biodiversity information for legislation, conservation, and management (Rydén et al., 2020). In hyperdiverse countries like the ones comprising the Central American area, this lack of informed regulations and the laxity with which transnational extractive projects are treated appear to be elements that allow continuous unregulated resource exploitation. If we follow the logic of the state capture phenomenon, the lack of scientific production regarding biodiversity could account for economic interests that would be affected by environmental protection measures. These contexts are not aligned with the worldwide call for global priorities, which includes generating an effective information basis of biodiversity distributions for safeguarding biodiversity and ecosystem services (Meyer, C. et al., 2015).

When comparing science production on biodiversity topics in Central America, there is a noticeable gap in the total amount of peer-reviewed scholarly works, patent development and work citations from the Northern countries (Guatemala, El Salvador, Honduras and Nicaragua) versus those from the South (Costa Rica and Panama). Costa Rica and Panama produce almost five times more scholarly works than their neighbors, being carried mainly by domestic researchers and institutions. On the other hand, the scholarly production from the rest of the region is executed mostly by international institutions whose first authors are commonly foreign researchers. The amount of georeferenced species occurrence records in natural collections, herbaria, and biological databases follows the same production tendency.

Over the last decades, this territory and its populations have been deeply affected by many tropical storms, hurricanes and other natural phenomena that evidentiate the region's vulnerability to climate change (Magrin et al., 2014; Hagen et al., 2022). This has been partially due to the lack of proper legislation that addresses forest coverage, biodiversity conservation, infrastructure and territorial ordering. How the state apparatus has been historically shaped and the way it operates through its judicial, executive and legislative branches, has a direct impact on what happens to the populations and species that inhabit these territories.

There is a historical and political explanation for these regional discrepancies, which we will attempt to unravel throughout the article using a bibliometric analysis and Bio-Dem –open-source software that compares species records and democratic variables per country– (Zizka et al., 2021). This paper aims at presenting a brief historical analysis from 1980 to 2020 of Guatemala, El Salvador, Honduras, Nicaragua, Costa Rica, and Panama^1^, to foreground how the democratic shifts throughout the years have had an impact on scientific mobility, biodiversity research, and species conservation records. Civil wars, coup d'etats, revolutions, dictatorships, migration waves, and international interventions are all events that have molded these countries' legislative frame and, therefore, their biodiversity conservation guidelines and science production.

## Methods

To understand the connections between biodiversity research production and the political framework of Central America, we opted for two different strategies. The first one was to use Bibliometrix to map scientific production through publications dealing with biodiversity; and the second one was using the Bio-Dem tool to explore how the number of species records per country can be related to three democratic variables: freedom of expression, political corruption, and polyarchy. Consequently, we examined how the historical shifts of such variables by country can explain the phenomena of state capture in the region and its impact on science development.

The historical milestones that define the traits of democracies in Guatemala, El Salvador, Honduras, Nicaragua, Costa Rica, and Panama were selected by means of a bibliographical revision centered in institutional documents, legislative proposals, newspaper articles, history books and peer-reviewed historical papers. Special attention was given to electoral processes, belligerent national conflicts, migratory waves, and natural phenomena, events that could account for the prominent fluctuations in the number of publications dealing with biodiversity topics per country, the differentiation of species records per country, and the links between local and international research institutions. It is important to mention that two of this paper authors are Central American with international graduate degrees, which allows for an insider's perspective on the regional history and the brain drain tendency.

### First Strategy: Data Collection and Bibliometric Analysis

To explore the amount of research on biodiversity being carried in Central America, we compared the scientific production on biodiversity subjects from 1980 to 2020 for each country, assessed through a science mapping analysis using Bibliometrix (open-source R-tool package) (Aria and Cuccurullo, 2017) and VOSviewer (1.6.18) (van Eck and Waltman, 2010). The bibliometric analysis strengthened the theoretical argument linking the three selected democracy variables, as proxies to the state capture phenomenon, and biodiversity research advances.

The data set was built through the Web of Science (WoS) indexing database accessed on January 31st, 2022. We opted for using WoS for its older trajectory in comparison to new–comer databases like Scopus. Given that our historical approach dealt with democratic shifts from 1980 to 2020, WoS was the best choice. There are multiple studies comparing these two databases, for our case, WoS presented more entries in environmental and biological sciences (Zhu and Liu, 2020). We built a search query using terms in titles, abstracts, and topics related to “biodiversity.” The search was refined by using only peer review publications in English, conference communications were excluded.

The next Boolean search query was used: TI = (Country) OR AB = (Country) OR TS = (Country) AND [TS = (biodiversity OR “biological diversity” OR Specie^*^ OR “species diversity” OR “species richness” OR “genetic diversity” OR ecosystem^*^ OR “invasive species” OR “endangered species” OR “conservation biology” OR “biodiversity conservation” OR biogeography OR “new species” OR taxonomy OR phylogeny OR “landscape ecology” OR “landscape”)] Timespan = 1980–2020. In WoS, “TI” = title, “AB” = abstract and “TS” = topic, relates to keywords, abstract, title, and keywords in this field. Documents in the fields of medicine, anthropology, archeology, business, economics, and social sciences were excluded. We validate our search queries for each country by reviewing the 50 most relevant entries and confirm that they suited the subject of biodiversity.

For the bibliometric analysis, the retrieved data was classified in four decades from 1980 to 2020 for each Central American country. It included the number of publications per country,^2^ top publishing country vs. local country number of publications, the top three publishing institutions, and the top five publishing authors (Table 1). All the authors appearing in the publications were counted. The top five authors were mapped in order to foreground their origin and scientific career, giving more attention to the affiliations for understanding the brain gain/drain process. The non-Central American top authors with a Central American institution affiliation were considered as part of the brain gain process in the region. The latter phenomenon, however, presents a much lower occurrence. The dataset used for the analysis is available in the FigShare repository—link in the Supplementary Materials section of the article.

**Table 1:** Bibliometric analysis of scientific production in biodiversity topics per decade from 1980 to 2020 in Central America.

**Decade**	**Country**	**Number of publications**	Top publishing country vs. local^*^	**Top publishing institutions**	Top publishing authors^**^
1980–1989	Guatemala	206	USA (46), GT (1)	(1) University of Wisconsin-Madison (2) North Carolina State University (3) University of Florida	David B. Wake Robert F. Martin Jack C. Schuster **(+)** David W. Greenfield Jonathan A. Campbell
	El Salvador	50	USA (10), SV (1)	(1) University of Costa Rica (2) University of Colorado (3) Shimane University	Jimmie C. Skinner Peggy S. Stanfill William E. Collins Hugo Hidalgo **(CA)** John S. Garth
	Honduras	108	USA (21), HN (3)	(1) York University (2) University of Florida (3) University of Costa Rica	Larry David Wilson James R. McCranie Martin Kellman John Hudson **(+)** Kenneth L Williams
	Nicaragua	76	USA (19), NI (1)	(1) Texas Tech University (2) University of Montana (3) University of California Berkeley	Egbert W. Pfeiffer Alison G. Power Curtis W. Sabrosky James E. Henrich Grady L. Webster
	Costa Rica	584	USA (142), CR (43)	(1) University of Costa Rica (2) University of Florida (3) University of California Berkeley	Daniel H. Janzen **(+)** Allen M. Young **(+)** Steven F. Oberbauer **(+)** Boyd R. Strain Gordon W. Frankie **(+)**
	Panama	634	USA (175), PN (42)	(1) Smithsonian Institute (2) University of Illinois (3) University of California Los Angeles	Henk Wolda **(+)** Carol K Augspurger **(+)** Russell Greenberg **(+)** David W. Roubik **(+)** Howard A Christensen (+)
1990–1999	Guatemala	324	USA (52), GT (5)	(1) National Autonomous University of Mexico (2) United States Department of Agriculture (3) University of Texas	Jonathan A. Campbell Gerald A. Islebe Eric N. Smith Jack C. Schuster **(+)** David B. Wake
	El Salvador	51	USA (10), SV (1)	(1) University of Colorado (2) National Autonomous University of Mexico (3) Louisiana State University	James R. McCranie Larry David Wilson W. E. Clark Cuauhtemoc Deloya A. Gomez-Sal **(CA)**
	Honduras	210	USA (36), HN (2)	(1) University of Florida (2) University of Connecticut (3) North Carolina State University	James R. McCranie Larry David Wilson David B. Wake David Lentz Janet W. Reid
	Nicaragua	151	USA (21), NI (2)	(1) University of Michigan (2) University of Maryland (3) Smithsonian Institute	Douglas H. Boucher John Vandermeer Amy Pool Francisco Collantes Ivette Perfecto
	Costa Rica	1137	USA (267), CR (67)	(1) University of Costa Rica (2) University of Florida (3) University of Miami	David B. Clark **(+)** Robin L. Chazdon Robert Lucking Deborah Clark **(+)** Manuel R. Guariguata
	Panama	831	USA (235), PN (125)	(1) Smithsonian Institute (2) University of Panama (3) Princeton University	Stephen P. Hubbell **(+)** Richard Condit **(+)** Robin B. Foster **(+)** Eldredge Bermingham **(+)** Klaus Winter **(+)**
2000−2009	Guatemala	655	USA (190), GT (19)	(1) National Autonomous University of Mexico (2) Autonomous University of San Carlos of Guatemala (3) University of Florida	Alejandro Estrada Enio B. Cano **(CA)** Jonathan A. Campbell Swen C. Renner David F. Whitacre
	El Salvador	191	USA (19), SV (6)	(1) University of Kansas (2) National Autonomous University of Mexico (3) University of El Salvador	Oliver Komar **(+)** V. Ernesto Méndez **(CA)** Bert Kohlmann **(+)** Alan S. Robinson David d Dame
	Honduras	413	USA (123), HN (4)	(1) University of Florida (2) Louisiana State University (3) National Autonomous University of Mexico	James R. McCranie Larry David Wilson Marco A. Zambrano David L. Anderson Josiah H. Townsend **(+)**
	Nicaragua	471	USA (109), NI (26)	(1) National Autonomous University of Mexico (2) Tropical Agricultural Research and Higher Education Center of Costa Rica (3) Central American University	Axel Meyer Jeffrey K. McCrary **(+)** Per Christer Oden Benigno González-Rivas **(CA)** Ivette Perfecto
	Costa Rica	2,820	USA (891), CR (354)	(1) University of Costa Rica (2) National Autonomous University of Mexico (3) National Institute of Biodiversity of Costa Rica	Daniel R. Brooks Daniel H. Janzen **(+)** Jorge Cortés **(CA)** Florencia Montagnini **(+)** David B. Clark **(+)**
	Panama	1,982	USA (730), PN (347)	(1) Smithsonian Institute (2) National Autonomous University of Mexico (3) University of Panama	Eldredge Berminghan **(+)** Hector M. Guzman **(CA)** Elisabeth K. V. Kalko **(+)** Richard Condit **(+)** S. Joseph Wright **(+)**
2010–2020	Guatemala	1,233	USA (366), GT (145)	(1) National Autonomous University of Mexico (1) Autonomous University of San Carlos of Guatemala (3) University of the Valley of Guatemala	Armando Cáceres **(CA)** Antje Schwalb Antonio Santos-Silva Liseth Pérez **(CA)** Danilo Alvarez **(CA)**
	El Salvador	372	USA (83), SV (43)	(1) University of El Salvador (2) National Autonomous University of Mexico (3) University of Costa Rica	Michael J. Liles Enrique Barraza **(CA)** Jeffrey A. Seminoff José D. Pablo-Cea **(CA)** Juan J. Morrone
	Honduras	862	USA (274), HN (70)	(1) National Autonomous University of Honduras (2) National Autonomous University of Mexico (3) University of Costa Rica	Merlijn Jocque James R. McCranie Joslah H. Townsend **(+)** Gustavo Fontecha **(CA)** Manfredo A. Turcios-Casco **(CA)**
	Nicaragua	972	USA (238), NI (77)	(1) National Autonomous University of Mexico (2) University of Konstanz (3) University of Costa Rica	Axel Meyer Kathryn R. Elmer Eva Harris Gonzalo Machado-Schiaffino Julián Torres-Dowdall
	Costa Rica	5,702	USA (1461), CR (1,120)	(1) University of Costa Rica (2) National Autonomous University of Mexico (3) Costa Rica Institute of Technology	M. Alex Smith **(+)** Winnie Hallwachs **(+)** D. Monty Wood **(+)** Daniel H Janzen **(+)** A. J. Fleming **(+)**
	Panama	3,619	USA (1266), PN (892)	(1) Smithsonian Institute (2) University of Panama (3) University of Costa Rica	S Joseph Wright **(+)** Benjamin L. Turner **(+)** Meike Piepenbring **(+)** Stephen P. Hubbell **(+)** Azael Saldana **(CA)**

* *Including the number of publications per country*.

** *(+) Authors with local affiliation; (CA) Authors born in Central America*.

The global data set (1980–2020 for all countries) consisting of 16,304 entries was also exported to the program VOSviewer (1.6.18) to create network visualization maps for the most influential countries, institutions, sources (journals), and terms in the Central American biodiversity science production. The strength of every node and its associations with the other elements in the network was presented as Total Link Strength (TLS) which is given in VOSviewer consequently by mapping research activity of the selected data set. The TLS is proportional to the extent of a specific node and its relationship with the other nodes, where a higher TLS value indicates greater collaboration, number of occurrences, and influence in the network. The threshold used for every map is explained in figures descriptions (**Figures 2**, **3**; Supplementary Figures S1, S2).

### Second Strategy: Exploring Species Records Data and Socio-Political Variables Through Bio-Dem

In order to infer the amount of research on biodiversity being carried in Central America and how it is linked to the democratic environment of the region, we opted for using Bio-Dem (open-source software, www.bio-dem.surge.sh). This tool allowed us to explore the relationship between Central American species occurrence records from the Global Biodiversity Information Facility (GBIF) (www.gbif.org) and the region's political framework from the Varieties of Democracy (V-Dem) database (www.v-dem.net) from 1960 to 2020 (Coppedge, 2020; Zizka et al., 2021). Geo-referenced species occurrence records deposited in GBIF have become crucial for biodiversity research and data modeling (Feldman et al., 2021), while V-Dem is the world's largest database dedicated to the collection and conceptualization of democracy data (Coppedge, 2020).

The availability of species records is linked to geographic accessibility, local investment in research, and political contexts (Meyer, C. et al., 2015; Daru et al., 2018; Eichhorn et al., 2020; Rydén et al., 2020). Therefore, the Bio-Dem software accesses the species record per year by country and allows for the user to relate them to 13 socio-political variables, which were postulated for having an impact on species occurrence record availability (Zizka et al., 2021).

**Figure 1:**
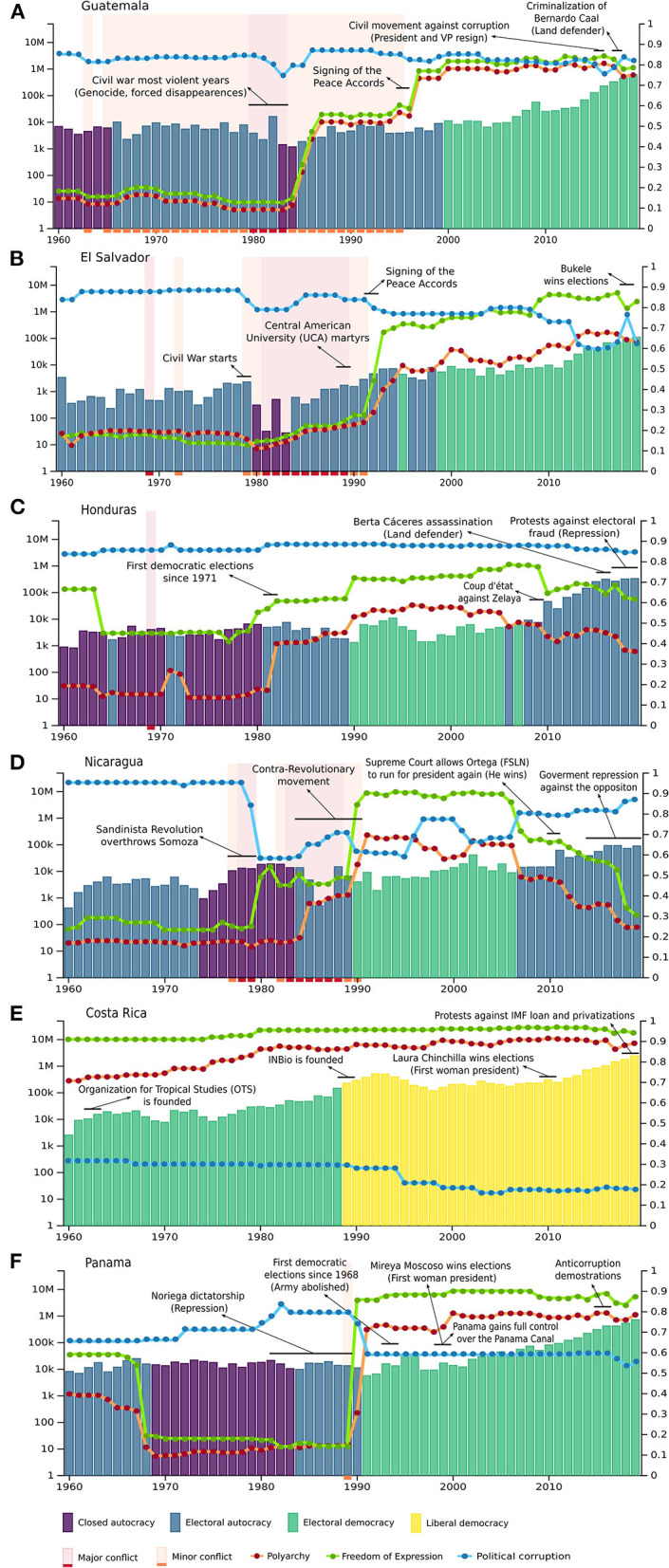
Number of species occurrence records and socio-political variables between 1960 and 2020 in Central America (by Bio-Dem). The three socio-political variables that were considered are (1) Freedom of expression (green), (2) Political corruption (blue), and (3) Polyarchy (red). Polyarchy refers to what degree a government selects its executive and legislative through popular elections (electoral democracy). Bar colors indicate political regime type, also showing minor and major conflict periods and key historical events for each country. In the case of Guatemala, Bio-Dem did not recognize the genocide as a major conflict, therefore we added the red background in the beginning of the 1980s when the genocide was executed. Note the logarithmic scale for the left y-axis corresponding to the species record number. The right y-axis corresponds to the chosen socio-political variables index.

We analyzed species record occurrence data and its possible links to political environments for each Central American country (Figure 1). The three socio-political variables that were considered for our analysis are (1) Freedom of expression, (2) Political corruption, and (3) Polyarchy^3^ (electoral democracy). Exploring these three elements of the Central American democracies will shed light on weak democratic transitions leading to a state capture phenomenon. There is a political and historical explanation for the gaps in species records in Central America which we will attempt to explain throughout this article

## When Science Production Meets Democracy (OR the Lack of IT)

Central America's democratic transition began in the 1980s with the *Esquipulas Agreement* (1986 and 1987), which sought to advance peaceful resolution of the regional conflicts and to promote economic and political cooperation between the isthmus countries (Sistema de Integración Centroamericana, 2022). This took place in the frame of the end of the Cold War and the fall of the Berlin wall in 1989. The ideological and geopolitical tensions embedded in the communism-capitalism debate were deeply felt in Central America. Therefore, when it started to seemingly decrease its intensity, it impacted the region's political forces configuration, which enhanced the possibilities of transitioning to what, in appearance, would look like a democratic era. However, as shown in Figure 1, the democracy indicators of the last decades highlight the political instability which has affected the chances of establishing strong research spaces in Central America, especially in those societies with a history of warfare, violence, and unstable electoral mechanisms. These conflicts were more acute in the region's Northern countries.

The internal conflict in Guatemala (1960–1996); the civil war in El Salvador (1979–1992); the overthrowing of the Somoza dynastic dictatorship in Nicaragua (1933–1979) and its subsequent contra revolutionary movement (1980–1991); Honduras having its first democratic elections in 1981 since 1971; and Panama undergoing the Noriega dictatorship (1981–1989), determined the last few decades of the twentieth century for the region and framed the chances of rigorous scientific production initiatives. While in 1989 Costa Rica created its local biodiversity think tank *InBio* (*Instituto Nacional de Biodiversidad*) in El Salvador eight Central America University (UCA) workers were assassinated by the regime, and Panama was having its first elections under US tutelage after the dictatorship (Figure 1). This is also reflected in the amount of science production and species records in the region. Political instability, apart from driving away investment opportunities, has obstructed inter-institutional science collaboration processes.

Renowned academics, thinkers, and social leaders were assassinated or disappeared during the guerilla war in El Salvador and Guatemala, internal conflicts that were supported by the US Government under the argument of the communist threats (Handy, 1984; Romano, 2012). These losses took a toll on the scientific population and emergent initiatives that would promote investments in research institutions that could guide data-based political and economic decisions. History itself was being contested by the powers involved in the decades-long conflicts in these countries, which impacted science production in the 1980s and 1990s, as shown in Table 1. Only after 2000, local institutions have had an influence on the research being carried on biodiversity topics in the region's Northern countries, which could be a byproduct of the democratic transition and how it enabled new funding influxes.

The democratic transition was supposed to reshape the State apparatus, integrating different sector interests: economic elites, high military stakeholders, big land-owners, and social minorities (laborers, women, indigenous, and Afro-Latin Central American populations). It would promote the redistribution of power quotas and the prioritization of resources investments. However, as expected after years of conflict and a history of colonial-cut state institutions, this process was rather a re-branding of the same former policies, leading to a deepening of structural inequalities with the entrance of neoliberal global economic policies promoted by Western countries. Said policies set the ground for the development of the state capture phenomenon worldwide, emerging strongly in the weak Central American democracies. “Civil war raged in Central America throughout the 1970s and 1980s and even into 1990s. It led to the deaths of at least 300,000 people, the vast majority of whom were killed by the military and/or right-wing hit squads. War produced between 1.8 and 2.8 m refugees. War also devastated the economies of El Salvador, Guatemala, and Nicaragua” (Lehoucq, 2014, p. 144). Intellectual exiles were amongst the population whom, to protect their lives, sought asylum as refugees elsewhere. Thus, becoming part of one the most significant diaspora of Central American thinkers of the last century.

The Washington Consensus in the 1990s, deemed as a neoliberalism starting point, promoted the so-called modernization of the State. These policy guidelines pushed for more market participation in social and economic matters (liberalization) resulting in the privatization of national services (Hernández Mack, 2010), and on a more pronounced shift toward extractivist concessions (mining, monocropping, irregular logging, hydroelectric dams). Such concessions had the tendency to operate through state capture mechanisms such as fiscal evasion, bribes and political lobbying, financing political campaigns, judicial capture and violation of labor, cultural and environmental rights. The latter led to evictions, repression, and the acute affectation of the region's ecosystems and biodiversity. The new international guidelines became requirements to apply for loans and funding from the International Monetary Fund (FMI) and the World Bank. This economic context along with the traits of young post-wars democracies, resulted in an economic crisis heavily felt by the working and middle class, and seized by local economic elites: “Though these policies curbed inflation and eventually resulted in slight economic growth, they inevitably pummeled the poor majority” (Walker and Wade, 2011, p. 97).

These democratic transitions and economic liberalization posed major challenges for the region's scientific production. The weakened institutionality—including research spaces—depended largely on international collaborations to function. Despite all Central American countries having a hyperdiverse landscape and being relatively small with large population density (**Table 5**), the amount of research on the subject does not reflect such reality. It is not the natural resources in a country nor the size of its population which determines how much research is being carried on the matter, but the socio-political context. The following section presents a thorough exploration of how the scientific production in the region has been affected by its convulsed history.

## Scientific Production on Biodiversity Topics in Central America

Bio-Dem and bibliometrics were used to explore possible links between historical democratic variables and the availability of biodiversity data, species records, and scientific production in Central America. The search was based on the publications addressing biodiversity science production in each Central American country from 1980 to 2020. The following entries were obtained per country: El Salvador (538), Honduras (1,059), Nicaragua (1,105), Guatemala (1,701), Panama (4,531), and Costa Rica (7,370). There is a considerable growth rate in every decade being the most productive years from 2000 to 2020 (Table 1).

When comparing the number of publications in Central America, there is a noticeable gap between the Northern countries (Guatemala, El Salvador, Honduras, and Nicaragua) versus those from the South (Costa Rica and Panama). The amount of georeferenced species occurrence records in natural collections, herbaria, and biological databases follows the same production tendency (Figure 1). Also, the democracy indicators vary per country throughout the years. In the Southern countries, the polyarchy index is higher than in the Northern ones, a manifestation of Costa Rica and Panama's more stable democracies in comparison to the ones in Guatemala, El Salvador, Honduras, and Nicaragua. The differences in the freedom of expression and political corruption values between Northern and Southern Central America also reflect a history of political convulsion and the inadequate structural arrangements that resulted from the region's democratic transition.

Costa Rica and Panama produce almost five times more scholarly works in biodiversity topics than their neighbors, being carried mainly by domestic researchers (local and foreign authors with an affiliation to a local institution). It is necessary to notice that in the first two decades (1980–1999) the research conducted in the Northern countries was led by foreign researchers that did not have an affiliation to a local institution. The top publishing and most influential country in the science production on biodiversity topics of all time in Central America is the United States (US), which is not a surprise given the extent of their funding programmes and the history of interventions in the region.^4^

**Figure 2:**
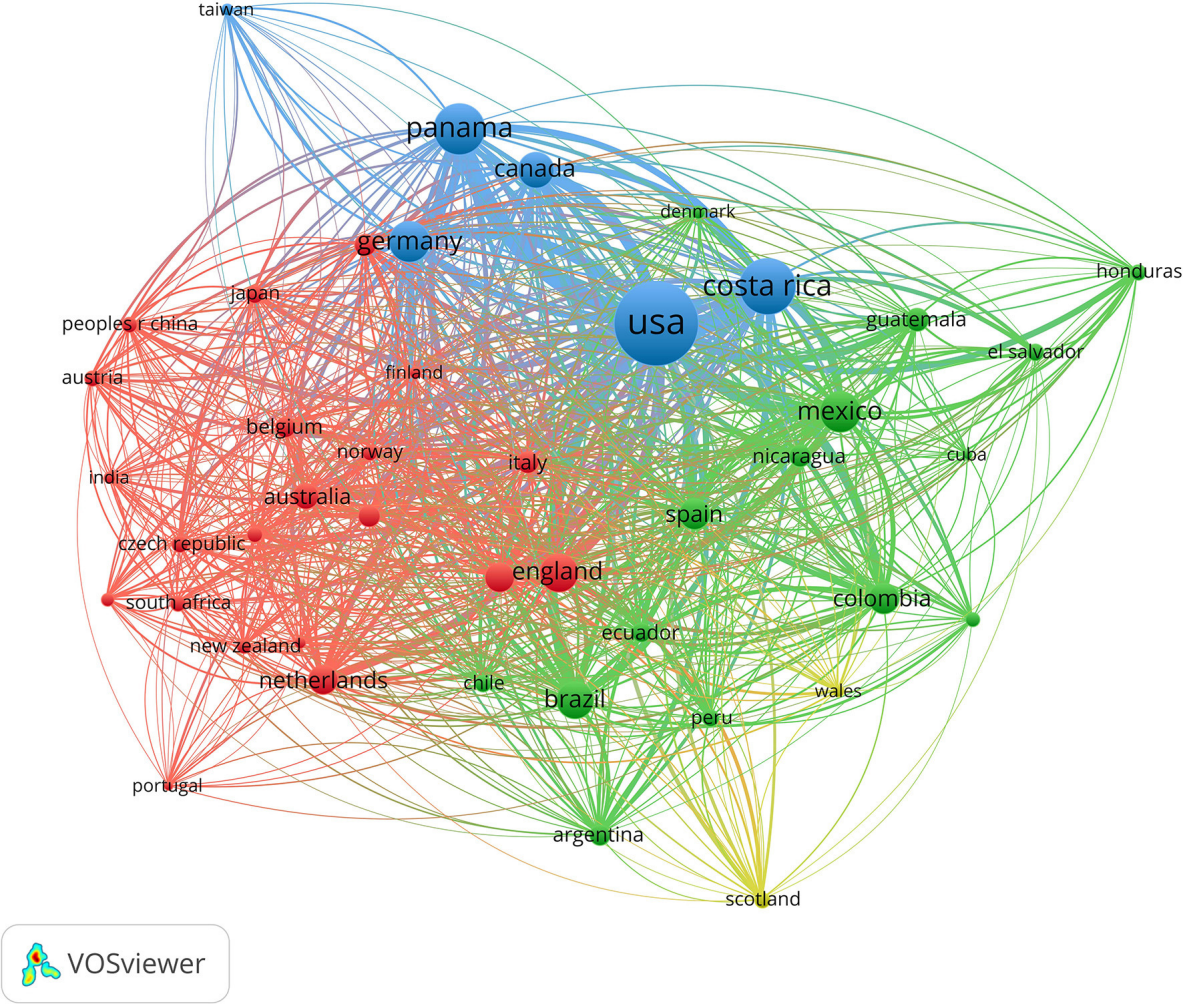
Network visualization map of international research collaboration among top active countries in biodiversity research in Central America. A threshold of at least 30 co-authored publications was applied. The size of the node and connecting line is proportional to the influence of the node in the network (number of publications and collaborations). Color indicates the relatedness of each cluster (by VOSviewer 1.6.18).

Panama and Costa Rica are the only two Central American countries with long-running participation in international scientific consortiums as observed in the node size in Figure 2. The construction of the Panama Canal under US administration at the beginning of the 1900s led to the creation of the Smithsonian Tropical Research Institute (STRI) in Panama. In the case of Costa Rica, in 1963 the Organization of Tropical Studies (OTS) was founded in cooperation with US scientists [Organization for Tropical Studies, 2021; Instituto Smithsonian de Investigaciones Tropicales (STRI), 2022]. These partnerships promoted brain gain processes in both countries making foreign researchers establish local affiliations. A great example is La Selva Research Station in Costa Rica which also gave local researchers the possibility to liaise with graduate biodiversity schools. These kinds of interactions and opportunities, limited as they are to having international research counterparts, explain one path the Central American scientific diaspora follows in order to find research opportunities.

We observed that only after the year 2000, science production increased not only in the total amount of publications but also in the participation of local institutions and researchers. This could be partially explained by the seeming improvement of the democracy indicators shown in Figure 1. A democratic and stable political environment would attract more investment of all kinds, science production being one of them.

A wider overview of collaboration among other countries with Central America is shown in Figure 2. The network analysis was created through VOSviewer, considering co-authored publications and country of affiliation. To refine the collaboration level, a threshold of at least 30 co-authored^5^ publications was applied. The TLS related to each country was highlighted proportionally to the size of the corresponding influence in the network, document frequency, and their number of citations (Table 2). Three main clusters of countries emerged.

**Table 2:** Top ten publishing countries in biodiversity topics in Central America.

**Rank**	**Country**	**Frequency**	**C/D**	**TLS**
1st	United States	6,455	28.3	4,652
2nd	Costa Rica	2,354	15.0	2,143
3rd	Panama	1,264	34.5	1,745
4th	Mexico	1,090	12.8	1,174
5th	Germany	923	24.9	1,135
6th	England	629	28.3	1,040
7th	Brazil	885	14.1	932
8th	Canada	721	29.0	914
9th	Colombia	405	21.8	667
10th	Spain	460	17.1	648
14th	Guatemala	265	15.7	391
17th	Nicaragua	198	18.3	345
26th	El Salvador	130	11.4	193
28th	Honduras	153	9.7	190

*C/D, number of citations per document*.

*TLS, total link strength in the network analysis*.

The US showed the highest level of influence in the network (4,652), followed by Costa Rica (2,143), Panama (1,745), and Mexico (1,174). In particular, US authors collaborated on biodiversity subjects with Costa Rican, Panamanian, German and Canadian researchers, respectively. This cluster shows that there is a link between the US and other Central American research institutions, being Honduras and El Salvador the two Central American countries with the least links to international institutions. The second most prominent cluster is the one comprising Latin American countries, in which Mexico, Colombia, Spain (for historical reasons), and Brazil were the most influential countries. The third cluster is the European one, including China, and South Africa to a lesser degree of influence in the network. This link responds to economical and geopolitical connections among these countries, mostly because of science and development funding projects.

Documents published by Panamanian-based researchers ranked first in the number of citations per document (34.5) followed by those published by researchers from Canada (29.0), US (28.3) and England (28.3). This highlights the Panamanian brain gain process and the impact of the STRI on the region. The network also foregrounds the most common destination countries for the Central American scientific diaspora. The United States is the most frequent foreign destination, reflecting its geographical and historical closeness to the region. The network analysis also showed that Costa Rica expresses a high degree of influence in the science production of the Central American Northern countries. Within the region, Costa Rica tends to be the most frequent destination for internal scientific migration.

We created a network analysis of the most influential institutions and a threshold of at least 50 co-authored publications was applied (Supplementary Figure S1). The top most influential institutions/organizations for biodiversity science production in Central America were dominated by Smithsonian Tropical Research Institute-Panama-, University of Costa Rica, University of Florida-US-, and National Autonomous University of Mexico (UNAM) (Table 3). For Central America, when it comes to science production on biodiversity subjects, only Panamanian and Costa Rican institutions shared the same influence compared to those of foreign countries. The majority of the most influential institutions in regards to biodiversity science production were from the US.

**Table 3:** Top ten publishing institutions/organizations in biodiversity topics in Central America.

**Rank**	**Institution/Organization**	**Frequency**	**C/D**	**TLS**	**Country**
1st	Smithsonian Trop Res Inst	1,044	44.1	938	Panama/USA
2nd	University of Costa Rica	1,166	9.7	477	Costa Rica
3rd	University of Florida	369	30.7	221	USA
4th	Auto Nat Univ of Mexico (UNAM)	475	14.3	220	Mexico
5th	University of California Berkeley	178	36.6	170	USA
6th	University of Illinois	146	38.6	170	USA
7th	University of Panama	132	12.0	158	Panama
8th	McGill University	105	37.0	122	Canada
9th	University of California St Cruz	106	50.9	119	USA
10th	Cornell University	135	31.5	118	USA

*C/D, number of citations per document*.

*TLS,Total link strength in the network analysis*.

For the most active journals dealing with biodiversity literature, we used a 50-occurrence threshold for network analysis. The leading journals were Biotropica-US-, Ecology-US-, *Revista de Biolog*í*a Tropical*-Costa Rica-, and the Journal of Tropical Ecology-UK- (Table 4). It is remarkable that *Revista de Biolog*í*a Tropical* (a Spanish/English journal from the University of Costa Rica) was one of the most influential journals in the region (Supplementary Figure S2). These results support our vision of how stronger democracies allow political stability for education investment, therefore, science production.

**Table 4:** Top ten publishing journals in biodiversity topics in Central America.

**Rank**	**Journal**	**Frequency**	**C/D**	**TLS**	**Country**	H-index^*^
1st	Biotropica	458	36.8	1,981	USA	96
2nd	Ecology	214	96.7	1,544	USA	297
3rd	Revista de Biologia Tropical	1,290	7.5	1,198	Costa Rica	38
4th	Journal of Tropical Ecology	224	36.7	1,169	UK	85
5th	Journal of Ecology	85	120.8	970	UK	181
6th	Oecologia	155	60.1	769	Germany	196
7th	Zootaxa	851	6.4	763	New Zealand	87
8th	Conservation Biology	72	105.9	602	UK	222
9th	Ecological Applications	79	60.9	473	USA	213
10th	Biological Conservation	85	50.3	444	Netherlands	199

*C/D, number of citations per document*.

*TLS, Total link strength in the network analysis*.

* *Source: Scopus*.

**Figure 3:**
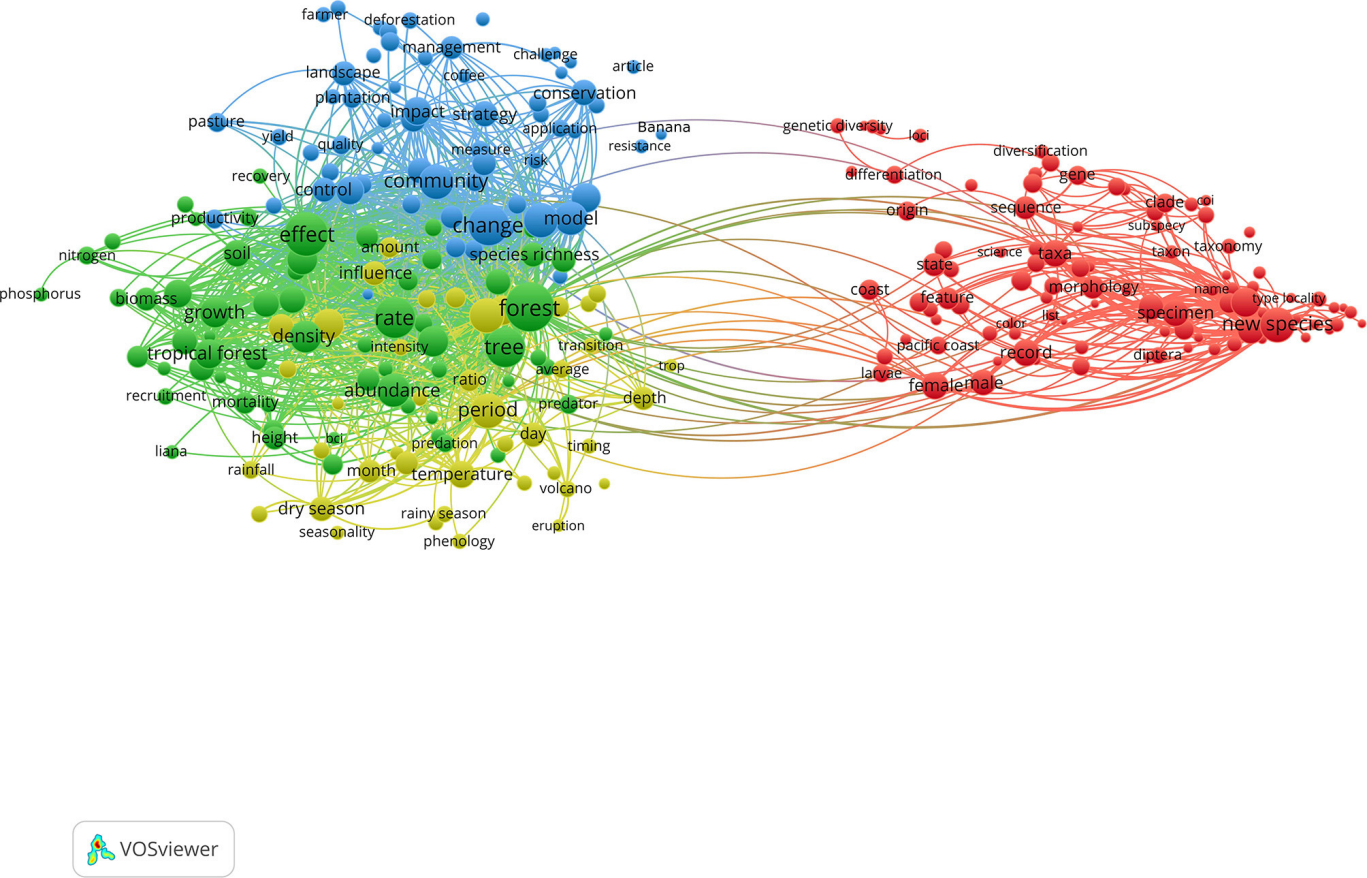
Network visualization map of terms in title and abstract fields of documents in biodiversity topics in Central America. Minimum occurrences of each term was 100. Nodes with the same color represent a cluster of related terms (by VOSviewer 1.6.18).

The most frequently used terms in the titles and abstracts of biodiversity scientific production in Central America were mapped applying a threshold of 100 occurrences. The outcome was the emergence of 265 words that were distributed in four clusters (Figure 3). The first cluster (red) included items focused on taxonomy, new species description, morphology, systematics, evolution, genetic diversity, entomology, and herpetology. The second cluster (blue) included terms focused on conservation, crops (banana, coffee), landscape, management, deforestation, and plantations. The third cluster (green) included items focused on ecology, soil science, forest dynamics, abundance, species richness. The fourth cluster (yellow) included terms focused on environmental sciences, temperature, dry season, rainy season, volcano, ecosystems transitions, phenology. It is worth mentioning that both of the crops most prominently studied in the publications—banana and coffee—are core commodities of Central America's gross domestic product. These were historically developed through the monopoly exerted by the US enterprise United Fruit Company in the early 1900s, and the German settlers and exporters. The growth of the banana and coffee exportation industry relied on labor force exploitation strategies, land evictions, and collusions between governments and foreign enterprises in the past century (Chomsky, 2021), as a preamble to the state capture phenomenon that emerged by the end of the century through neoliberal guidelines. It is also important to notice the influence that the United States Department of Agriculture (USDA) has had on the region, being one of the top publishing institutions for Guatemala in the 1990s (Table 1).

## Conservation and Climate Change on the Isthmus

Both the social and natural landscape in Central America suffered from the implementation of neoliberal policies and, in many cases, these two dimensions were affected at the same time. For example, despite Honduras ratified the International Labor Organization (ILO) Convention No. 169 in 1995, which recognizes the right of the populations to be consulted before extractivist concessions, over the last few years there has been a discussion surrounding a new legislative proposal that redefines the consultation guidelines giving the Government the final decision (International Labour Organization, 2017). This process has had the support of the Inter-American Development Bank (IDB) and the United Nations Development Program (UNDP) (Corea, 2018).

The vast majority of the extractivist projects approved in Central America have arisen social conflict due to the lack of sustainability, legal and social guidelines that could reduce the impact they inflict on the ecosystems and communities. However, such extractive industries have been able to modify laws, obtain environmental licenses and use the judicial representatives to imprison social leaders who oppose their unregulated industrial activities. In sum, they have implemented a corporate state capture strategy. For instance, the criminalization of the maya-q'eqchi' land defender Bernardo Caal in Guatemala who was sentenced in the midst of a high political corruption environment. In Honduras, as well, the indigenous lenca leader and land defender Berta Cáceres was assassinated for defending the Gualcarque River against a hydroelectric dam project (Figure 1). The democracy indicators might show high levels of freedom of expression in comparison to previous decades, and do not display dictatorships as a reality in Central America. However, the State structure that resulted from the democratic transition and was oriented by a neoliberal world agenda, has developed new legal ways of repression and coercion. The state capture phenomenon is not an illegal one, it rather uses legal strategies to promote private interests above public ones. In a region where traits of formal democracy coexist alongside practices and institutions that tend to be part of authoritarian regimes (Desmond Arias and Goldstein, 2010), the government becomes a corrupt mediator between transnational companies and the population. At the same time, refusing to support its decisions on trustworthy scientific production while cutting funding for academic institutions. Academic institutions are perceived as a menace in the context of political instability and lack of democratic guarantees due to their intellectual reflective capacity and their social mobilization potential. In Nicaragua, for example, over the last few months the Ortega regime has closed five different universities (AFP, 2022). This reality shows that, despite being a biodiversity hotspot containing unimaginable possibilities that could offer alternatives for everyday issues, Central American governments do not prioritize research on conservation^6^ nor social welfare.

It is important to highlight that climate change is one of the biggest threats to Central America's biodiversity, therefore, to its populations and ways of living. The overpopulation in the region's countries is correlated to the exploitation of its natural resources and the scarcity of their access. It is not a coincidence that the Northern countries, where the extractive industry is more developed and unregulated, are more overpopulated than the Southern ones. For example, El Salvador, which is the smallest country in the region, has a larger population than Costa Rica (Table 5). This is a direct threat to biodiversity in each country because larger populations overload the carrying capacity of Central American ecosystems.

**Table 5:** Funds assigned for research in Central America.

**Country**	**GDP% allocated to Research and Development, year (a)**	**No. of researchers per million people, year (b)**	**Area (Km^**2**^)**	**Population in millions (c)**	**Population density in Km^**2**^**
**Central America**					
Costa Rica	0.38 (2018)	345 (2018)	51,000	5,094,114	96.93
El Salvador	0.16 (2018)	71 (2018)	21,041	6,486,201	304.72
Panama	0.15 (2017)	39 (2013)	75,420	4,314,768	55.12
Nicaragua	0.11 (2015)	70 (1997)	130,370	6,624,554	48.53
Honduras	0.04 (2017)	35 (2017)	112,090	9,904,608	83.71
Guatemala	0.03 (2018)	13 (2018)	108,889	16,858,333	158.38
Core-countries^*^					
US	2.83 (2018)	4,412 (2017)	9,833,520	331,501,080	36.2
China^**^	2.14 (2018)	1,307 (2018)	9,596,961	1,410,929.36	149.7
UK	1.70 (2018)	4,603 (2018)	242,495	67,215,293	281.18

* *Included for comparison purposes*.

** *Without including Hong Kong or Macao Special Administrative Regions*.

Source:

*(a) World Bank (2019) https://datos.bancomundial.org/indicador/GB.XPD.RSDV.GD.ZS*.

*(b) World Bank (2021) https://data.worldbank.org/indicator/SP.POP.SCIE.RD.P6*.

*(c) World Bank (2019) https://data.worldbank.org/indicator/SP.POP.TOTL*.

In 1988, the United Nations (UN) created the Intergovernmental Panel on Climate Change (IPCC) to promote discussions and agreements that would mitigate the possible effects of environmental changes due to the effects of pollution of large extractive practices. By 1992, the IPCC—which was attended by all Central American countries—agreed to reduce carbon emissions from industrialized countries to stop global warming. In 1997, the Kyoto Protocol called for the reduction of emissions from industrialized nations, and once again all Central American countries signed the agreement. However, the core-countries'^7^ political and economic interests prevented the protocol from gaining traction until 2005.

“Between 1998 and 2010 Central America experienced a substantial increase in the recurrence of severe and extreme weather phenomena associated with climate change” (Stein, 2014, p. 64). The IPCC 2014 report remarked on the vulnerability of Central America in the next 50 years, demonstrating how climate change and high levels of deforestation (mainly in Guatemala, Honduras, El Salvador, and Nicaragua) could impact crops production, flooding/droughts, and the frequency of tropical infectious diseases (Magrin et al., 2014). In 2016 all Central American countries signed the Paris Agreement for a long-term temperature goal to keep the rise of global temperature below 2°C above pre-industrial levels. These international pacts should be a top priority in a region that, as explained, is highly vulnerable to climate change.

Further studies and systematic reviews of climate change-related risks for the region have identified contingencies associated with food insecurity, floods and landslides, water scarcity, epidemics, coral bleaching, tropical storms, erosion, and sea-level rise until 2070 (Hagen et al., 2022). These effects have been perceptible in the last years through natural and social phenomena, like hurricanes Eta and Iota that affected more than 7 million people and caused almost $7 billion (AON, 2020) in damage. Also forced migrations phenomena like the Central American Migrant Caravans, more than 7000 people traveling from Central American countries to the Mexico-US border searching for refuge and labor opportunities (Pradilla, 2019). The main cause is disaster displacement, human populations living in fragile ecosystems affected by floods or droughts or/and living in conflict-affected areas with hostile/violent groups like “maras” gangs or drug trafficking. The most affected populations are rural communities, indigenous people, Afro-Latin Central Americans, women, LGBTQIA+ and migrants (Magrin et al., 2014; Hagen et al., 2022).

## Understanding Scientific Diaspora in Central America

Latin American countries experience scarcity in science funding, such investment is considerably lower in Central America (Table 5). During the democratic transition period for the region, the national councils for science and technology^8^ were created: Costa Rica in 1990, Guatemala in 1991, El Salvador in 1992, Honduras in 1993, Nicaragua in 1995, and Panama in 1997. This means that before the 1990s Central American countries did not have clear research guidelines nor state funding for it. However, these institutions operate with very little porcentage of the national GDP that is assigned yearly to research and development, which is especially acute for Honduras and Guatemala both the countries with the lowest number of researchers per million people (Table 5).

Guatemala and El Salvador, the region's most populated country and the one with the highest population density per km^2^ respectively, have only one state university. For the entire region it is safe to say that most higher education institutions are private, adding up to the privatization of democracies' tendency, and commodifying the access to education. In Panama, the mean years of schooling per person is 10.2, while in Honduras and Guatemala it is 6.4 years (PNUD, 2018). These facts are the mere reflection of a deficient educational system, both in regards to quality and access, another outcome of systematic corruption and low democratic indicators (Figure 1). Some of the features that the Central American Northern countries share include “the precariousness of their higher education systems (and education in general), the institutional weakness of public bodies and governance relevant to science, technology and innovation and a private (industrial) sector disconnected from the greater national development project” (Bonilla and Serafim, 2021: 24).

The lack of support for STEM careers (Science, Technology, Engineering, Mathematics) from state institutions is one of the fundamental reasons for the underdevelopment of the region's science production (ídem). Advanced biodiversity research like genomics, transcriptomics, LiDAR technology, bioinformatics, and sampling big areas for instance, requires high financing. There is a lack of graduate schools awarding academic degrees in advanced biodiversity topics like Molecular Biology, Genetics, Advance Ecology, Soil Sciences, Geology, Oceanography, Marine Biology, Environmental Sciences, Forestry, Zoology, Botany, especially in Guatemala, El Salvador, Honduras, and Nicaragua.

Local science production tends to be invisibilized because most domestic professionals only have an undergraduate degree, which means that they are not familiarized with science communication nor peer reviewed publication processes. Scholarships and fellowships for undergrad and graduate students in these countries are almost non-existing, leaving little options to those who want to pursue a career in STEM. The continuation of academic training tends to be abroad, a defining reality of the brain drain phenomenon (Bonilla, 2018). Countries like Panama, for example, have developed repatriation programs (2010) with support from the IDB to foster the return of trained national scientists (Torres-Atencio, 2022). This contrasting scenario for science performance in Central America limits the local research scope in regards to international collaborations, events, publications, and the advance of science in general.

These barriers seem to be less frequent for core-country scientists (global North), to whom pursuing research in Central America (or Latin America) is more plausible than for domestic professors and students. Within the region, Costa Rica and Panama are the only countries with more specialized science research in biodiversity topics, making them a destination for the internal Central American scientific diaspora. This tendency follows what Chinchilla-Rodríguez et al. (2018) posed in their study on scientific mobility, where core-countries showed less collaboration ratios than periphery ones. These ties, as shown in our article, are highly resource-dependent in nation-state contexts of corruption and political instability. The core-countries have historically accumulated resources by means of extraction, colonialism and neo-colonialism, therefore they do not present an urge to have international funding. Their counterparts, on the contrary, are selected upon research interest. There is broad evidence of colonialism having a direct impact on diversity research (Rydén et al., 2020). It appears that species records availability and number of publications is tied to the author's origin. For example, in the Journal of Biogeography (Wiley), the number of publications correlates with the first and corresponding author's nationality. According to the decolonial analysis presented by Eichhorn et al. (2020), out of all the papers published in said journal, Central America ranks amongst the lowest publishing regions in the world.

Tracking scientific mobility in Central America, outside and within the region, remains a challenge. A few governamental databases for tracking local scientists' paths are available, but they have limitations on presenting clear data and statistics that could inform where local scientists are establishing their careers. The most frequent destinations of the Central American scientist diaspora are the US, Mexico, Western-European countries, and to a lesser extent other Global South destinations (Chinchilla-Rodríguez et al., 2018), which is also observed on the network analysis for the most influential countries in biodiversity research in Central America (Figure 2).

Latin America and the Caribbean are amongst the most unequal regions of the world [Comisión Económica para América Latina y el Caribe (CEPAL), 2022]. In the case of Central American scientists, taking into account the decades-long democracy instability, they are forced to overcome deep social, cultural and ethnic barriers. All of the advanced scientific production, not just on biodiversity topics, is expected to be produced in English in order to be published in renowned journals. In a region that lacks academic institutions and funding to promote research, local scientists struggle to cover the fees for international publications or even to access specialized and updated literature (Ciocca and Delgado, 2017). According to our analysis, less than half of the science production on Central American biodiversity is open-access. A gender gap bias is also reflected in the disparity of the number of authorship by women authors versus that from men (Table 1), which reflects a reality in which manuscripts submitted by men are more likely to be accepted (Valenzuela-Toro and Viglino, 2021). These disparities are accentuated when ethnicity and ableism are taken into consideration.

## Conclusion

The Central American region is highly vulnerable to climate change effects. Despite the isthmus countries having ratified several international agreements on the matter, their investment on research and science does not reflect it as a priority. Without a state policy oriented toward the promotion of researching institutions (grants, scholarships, fellowships, etc.), trained researchers and scientists that were privileged enough to access higher and specialized education are forced to migrate to pursue professional opportunities. This brain drain phenomenon hinders the development of science based solutions to the Central American countries' most pressing issues.

The low scientific production in Central America is not the result of lack of ability or talent, but rather the outcome of convulsed historical processes that weakened state structures and institutionality, and framed how the populations have reacted to ongoing conflict. This facilitated the capture of the state by local and international economic powers, determining how the resources are distributed through systemic corruption practices. As suggested, scientific production tends to be more prolific when democratic conditions are guaranteed. Despite the barriers encountered by the scientists of the region, the results demonstrate that the research production on biodiversity topics has been steadily increasing over the years. A higher production of biodiversity science was observable in Costa Rica and Panama in contrast to their Northern region neighbors, which again expresses how science advances are built upon strengthened and institutionalized research and stable democratic contexts that allow for collaborations and investment.

This analysis showed that the United States is the country with the tightest academic links to Central America (Figure 2) and its biodiversity research production. This country's historical interaction with the region has mostly been through economic/political intervention: the numerous decades of monopoly through the United Fruit Company since the beginning of the 1900s, the contra-revolutionary support in countries like Guatemala (1954) and Nicaragua (1980-1991) which halted democratic processes (Torres-Rivas, 2007), and the construction (1903–1914) and ownership of the Panama Canal by the US until 1999. Taking into consideration such history, the level of US influence on the science being produced about Central America's natural landscape leads to some broader reflections. The fact that most of the publications regarding Central American biodiversity throughout the analyzed decades came from US institutions with no links to local hubs perfectly exemplifies science extractivism, which has become an extension of modern colonialism (Eichhorn et al., 2020; Rydén et al., 2020; Zizka et al., 2021) that continues to deepen dependencies more than fostering collaborations amongst peers. The understanding of science production on biodiversity topics for environmental and social legislation has a direct link to democratic and historical processes.

Our data set for studying biodiversity science production in the region was based on the use of Bio-Dem as a platform that is fed solely by the data presented in the GBIF, and the V-Dem research project. There are other initiatives of citizen science platforms that could be taken into consideration, especially in regards to species records (Feldman et al., 2021). Our bibliometric analysis had some limitations that are worth pointing out. The entries in the WoS database only take into consideration peer reviewed works in English, framing the reach of our exploration. The fact that Spanish writing journals are not considered in WoS database, expresses the bias of science productions in non-English speaking countries. Another limitation was the country of origin of the publication in the bibliometric analysis, which was considered just by the main affiliation of the corresponding author(s) even though some of the other authors might have been from local countries.

Despite these limitations, our work still underpins tendencies that shed light on some of the challenges that scientific production and scientific mobility faces in Central America and their historical causes. Democracy, science, and conservation are core elements that go hand in hand and that need to be nourished in a region that struggles with extractive activities and the protection of life.

## Data Availability Statement

The original contributions presented in the study are included in the article/Supplementary Material, further inquiries can be directed to the corresponding author/s.

## Author Contributions

JM-M conceived and coordinated the study, carried out the bibliometrics and network analysis, and explored the Bio-Dem tool. RS led the historical and political analysis. JM-M and RS designed and drafted the manuscript. MZ and JB contributed to the edition and funding of the article. All authors read and approved the final manuscript.

## Funding

We acknowledge the financial support from the São Paulo Research Foundation (FAPESP—grant #2021/10319-0) and the National Council for Scientific and Technological Development (CNPq—grant #431226/2018-0). We also thank the Coordination for the Improvement of Higher Education Personnel of Brazil (CAPES) for the granted scholarship Ph.D. to JM-M.

## Conflict of Interest

The authors declare that the research was conducted in the absence of any commercial or financial relationships that could be construed as a potential conflict of interest.

## Publisher's Note

All claims expressed in this article are solely those of the authors and do not necessarily represent those of their affiliated organizations, or those of the publisher, the editors and the reviewers. Any product that may be evaluated in this article, or claim that may be made by its manufacturer, is not guaranteed or endorsed by the publisher.

## Footnotes

^1^This analysis did not include Belize on the account that it is a parliamentary constitutional monarchy following the British model after gaining independence in 1981, and not a presidential representative democratic republic, like the rest of the Central American countries which in the 80s were experiencing convulsion due to civil wars and dictatorships. There is also a cultural difference between Belize and the rest of the region's countries in terms of the language.

^2^The variable “country” for an entry is where the corresponding author(s) have their main institutional affiliation.

^3^Following Zizka et al. (2021), polyarchy is understood as “to what degree does a regime select its executive and legislative through popular elections, whether these elections are free and fair, and how widespread the right to vote is” (p. 2718).

^4^For example, the execution of the operation PBSuccess in Guatemala (1954) to overthrow the democratic president Jacobo Árbenz Guzmán, and supporting the contra-revolutionary movement in Nicaragua (1980–1991) (Torres-Rivas, 2007).

^5^The threshold of co-authored publications indicates the number of occurrences that the variable (country of affiliation, term, institution or journal) needed to have to be included in the network analysis. In comparison to other studies using VOSviewer like Sweileh (2020), we opted for a larger threshold, allowing for a more in-depth analysis.

^6^Some steps in that direction have been given by Costa Rica, El Salvador, and Honduras who forbid open-pit mining in 2011, 2017 and 2022 respectively.

^7^Following Wallerstein (2005) throughout this article, the authors will be using the terms “core-country” and “periphery-country”. This must not be perceived as a mere change in the terminology—developed vs in ways of development—, but rather implies a different analytical approach that's informed by the World-system theory and the Dependency theory. That is, the acknowledgment that the idea of development is outdated and presents a false illusion of linear transformations with one sole reaching point molded by industrialized countries. The core-periphery categories refer to unequal exchange dynamics and it places the emphasis on the analysis of historical systems and not on the nation-state category.

^8^Costa Rica is the only Central American country which has upgraded this institutional space to a Ministry of Science, Innovation, Technology and Telecommunications (MICITT).
