# Supplementary material for: Biodiversity Research in Central America: A Regional Comparison in Scientific Production Using Bibliometrics and Democracy Indicators
Source: Front Res Metr Anal. 2022 Jul 14;7:898818. doi: 10.3389/frma.2022.898818 (PMC9329674; doi:10.3389/frma.2022.898818)

***Supplementary Material***

Supplementary data. Our dataset for the bibliometric analysis is available at doi: 10.6084/m9.figshare.20069918. The global data set (1980-2020 for all Central America) consists of 16,304 entries: El Salvador (538), Honduras (1059), Nicaragua (1105), Guatemala (1701), Panama (4531), and Costa Rica (7370).

Figure S1 Supplementary Material. Network visualization map of the top influential institutions in biodiversity research in Central America. A threshold of at least 50 co-authored publications was applied. The size of the node and connecting line is proportional to the influence of the node in the network (number of publications and collaborations).


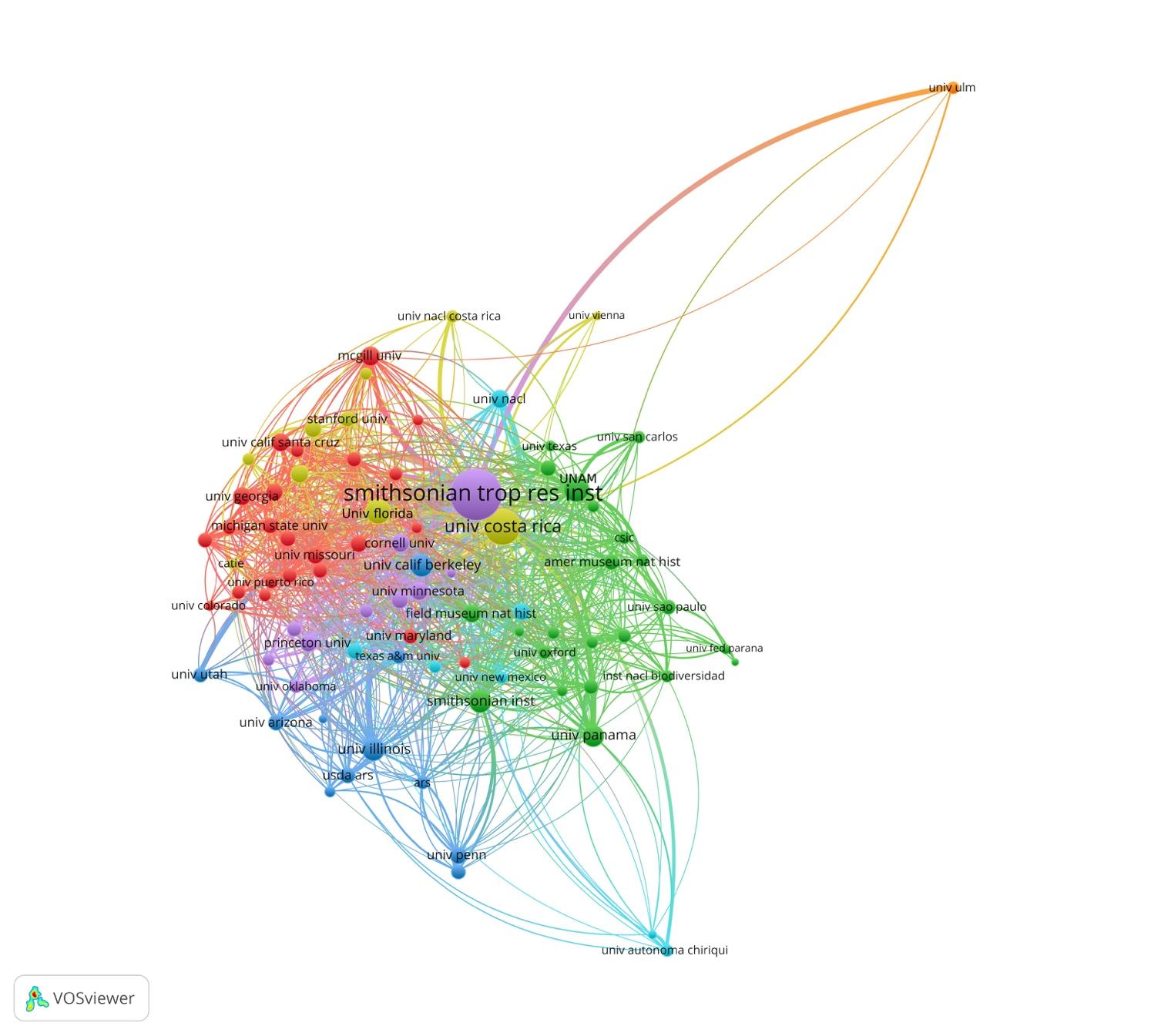


Figure S2 Supplementary Material. Network visualization map of the top influential journals in biodiversity research in Central America. A threshold of at least 50 co-authored publications was applied. The size of the node and connecting line is proportional to the influence of the node in the network (number of publications and collaborations).


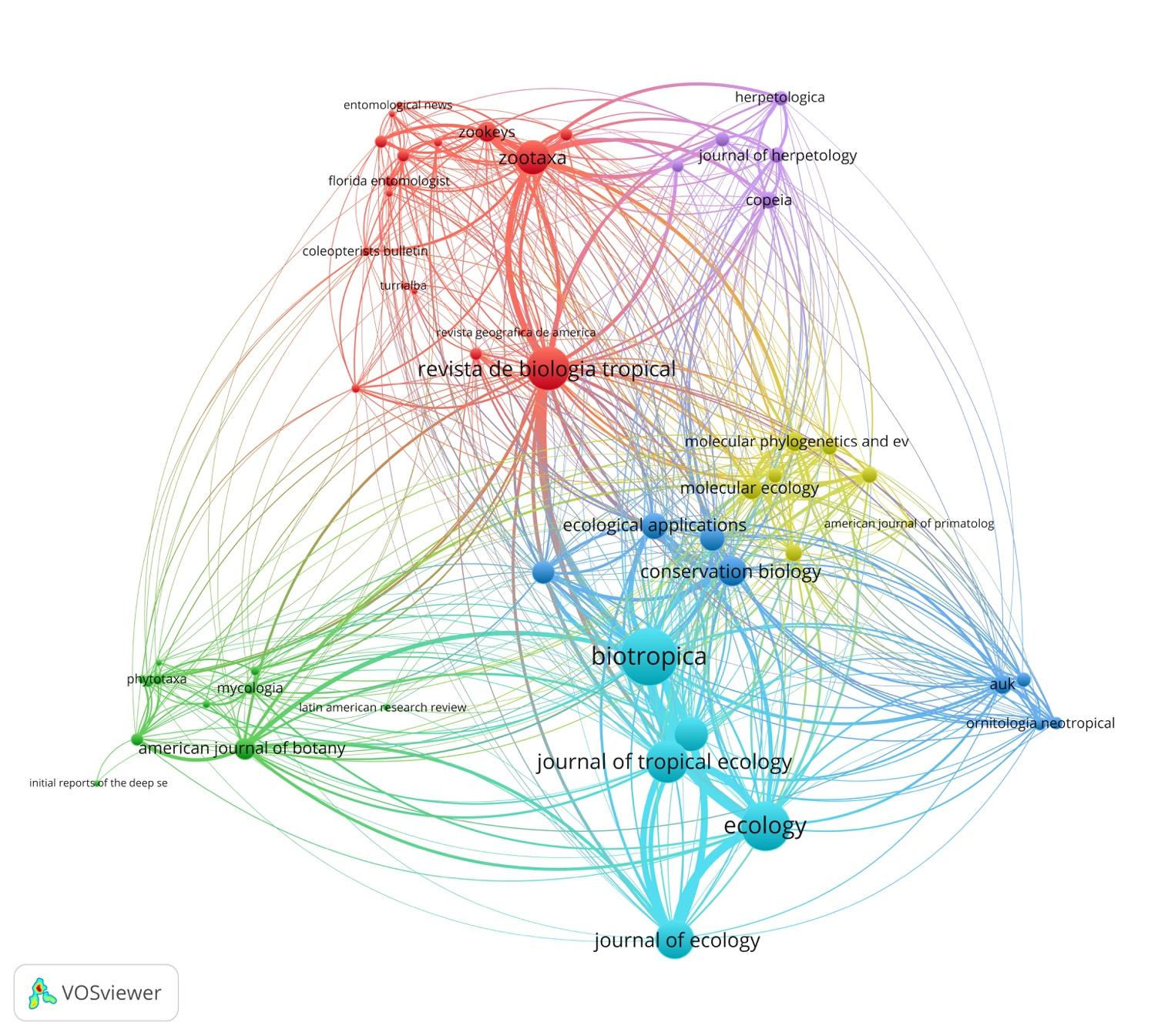

Supplement: Supplementary file 1 [file Data_Sheet_1.docx]
